# Opportunities and Obstacles for Providing Medical Education Through Social Media

**DOI:** 10.2196/15297

**Published:** 2019-11-27

**Authors:** Aimee Wilkinson, James Ashcroft

**Affiliations:** 1 Imperial College London London United Kingdom

**Keywords:** medical education, social media, innovation

## Abstract

Social media has infiltrated almost every sector of life, and medical education is no exception. As this technology becomes mainstream within society, an increasing number of health care students and professionals are using it for learning. Several important considerations for the risks of this technology are discussed here.

Social media has drastically altered how the world communicates. No longer a matter of science fiction, large quantities of public and private data can now be exchanged in minutes. A recent Journal of Medical Internet Research report examined the successes of the YouTube channel “Not Just a Medical Student,” which attempts to innovatively apply social media for the advancement of medical education [1]. As a recently graduated doctor and current undergraduate medical student who regularly use social media for medical education, we would like to share our perspectives on this report and future advances within this field.

Medical school curricula often lack teaching on leadership, teamwork, and innovation, all of which could be taught through social media. Despite saturated curricula, these elements are key to health care advancement and of particular importance when undertaking leadership or innovation roles after graduation. More broadly, the benefits of educational social media are clear. By overcoming geographical and time barriers, it allows students equal access to teaching and is associated with higher levels of student satisfaction [2]. Social media platforms have a further advantage over conventional communication methods, in that these online spaces are already accessed as part of users’ daily routines [2,3]. For example, it is estimated that 44.5% of medical trainees and 64.3% of medical students have active Facebook accounts [4].

The application of social media to medical education is not without risks. Professionals and patients alike are susceptible to false information, and such content can easily be distributed if time is not taken for validation and peer review. These strategies are further important to safeguard the professional reputations of individuals and institutions. Without considering regulation and the need for a clear distinction between personal and professional opinion, public perception of medical practice may be affected and patients could be dissuaded from choosing truly beneficial treatments. Lack of regulation further permits the spread of biased information, such as exaggerated or misleading claims from industries with financial interests. Finally, discussion of real cases risks physicians accidentally sharing personal information and thereby breaching confidentiality. A survey of 1600 health science staff and students found that the greatest barriers to educational social media use were concerns about policies and professionalism [3]; hence, training is required to address these concerns and prevent doctors from becoming liable for damages. Overall, a compromise on speed is warranted to ensure that all content adheres to guidance from regulatory bodies before publication.

Future work should address the lack of quantitative evidence to support claims that social media is an effective educational tool [5]. The presently used metrics, such as the number of likes, shares, and comments a post receives, require assessment to decipher why exactly an audience deems a video favorable or shareable, and research into whether these metrics are indicative of educational value is needed. We believe that social media is a powerful tool with the potential to improve medical education and the lives of patients worldwide. We thank the “Not Just a Medical Student” team for producing such innovative content and look forward to seeing how this field progresses.
